# Supplementary material for: Social Network Analysis of Agonistic Behaviour and Its Association with Economically Important Traits in Pigs
Source: Animals (Basel). 2020 Nov 16;10(11):2123. doi: 10.3390/ani10112123 (PMC7696858; doi:10.3390/ani10112123)
Supplement: Supplementary file 1 [file animals-10-02123-s001.pdf]

Table S1. The number of animals and number of couples involved in agonistic action in each pen.

Pen	No. of Animal per Pen	No. of Couples Involved in Agonistic Action in each Pen
1	13	34
2	15	26
3	12	10
4	15	36
5	13	30
6	11	4
7	11	9
8	11	5
9	11	1
10	12	2
11	12	6
12	12	12
13	10	21
14	10	14
15	13	22
16	13	13
17	12	17
18	9	22
19	11	17
20	11	17
21	11	17
22	11	8
23	11	12
24	11	13
25	12	7
26	12	9
27	12	5
28	11	3
29	10	4
